# Hsp27 Responds to and Facilitates Enterovirus A71 Replication by Enhancing Viral Internal Ribosome Entry Site-Mediated Translation

**DOI:** 10.1128/JVI.02322-18

**Published:** 2019-04-17

**Authors:** Xuelian Dan, Qianya Wan, Lina Yi, Jing Lu, Yang Jiao, Huangcan Li, Dan Song, Ying Chen, Hongxi Xu, Ming-Liang He

**Affiliations:** a Department of Biomedical Sciences, City University of Hong Kong, Kowloon, Hong Kong; b Guangdong Provincial Institution of Public Health, Guangdong Center for Disease Control and Prevention, Guangzhou, China; c School of Pharmacy, Shanghai University of Traditional Chinese Medicine, Shanghai, People’s Republic of China; d CityU Shenzhen Research Institute, Shenzhen, China; University of Texas Southwestern Medical Center

**Keywords:** CRISPR/Cas9, IRES, enterovirus A71, heat shock protein 27, hnRNP A1

## Abstract

Enterovirus 71 (EV-A71) is a human pathogen that causes hand, foot, and mouth disease (HFMD) and fatal neurological diseases, and no effective treatment is available. Characterization of key host factors is important for understanding its pathogenesis and developing antiviral drugs. Here we report that Hsp27 is one of the most upregulated proteins in response to EV-A71 infection, as revealed by two-dimensional gel electrophoresis-based proteomics studies. Depletion of Hsp27 by small interfering RNA or CRISPR/Cas9-mediated knockout significantly inhibited viral replication, protein expression, and reproduction, while restoration of Hsp27 restored such virus activities. Furthermore, we show that Hsp27 plays a crucial role in regulating viral internal ribosome entry site (IRES) activities by two different mechanisms. Hsp27 markedly promoted 2A^pro^-mediated eukaryotic initiation factor 4G cleavage, an important process for selecting and initiating IRES-mediated translation. hnRNP A1 is a key IRES *trans*-acting factor (ITAF) for enhancing IRES-mediated translation. Surprisingly, knockout of Hsp27 differentially blocked hnRNP A1 but not FBP1 translocation from the nucleus to the cytoplasm and therefore abolished the hnRNP A1 interaction with IRES. Most importantly, the Hsp27 inhibitor 1,3,5-trihydroxy-13,13-dimethyl-2*H*-pyran [7,6-*b*] xanthone (TDP), a compound isolated from a traditional Chinese herb, significantly protected against cytopathic effects and inhibited EV-A71 infection. Collectively, our results demonstrate new functions of Hsp27 in facilitating virus infection and provide novel options for combating EV-A71 infection by targeting Hsp27.

**IMPORTANCE** Outbreaks of infections with EV-A71, which causes hand, foot, and mouth disease, severe neurological disorders, and even death, have been repeatedly reported worldwide in recent decades and are a great public health problem for which no approved treatments are available. We show that Hsp27, a heat shock protein, supports EV-A71 infection in two distinct ways to promote viral IRES-dependent translation. A small-molecule Hsp27 inhibitor isolated from a traditional Chinese medicinal herb effectively reduces virus yields. Together, our findings demonstrate that Hsp27 plays an important role in EV-A71 infection and may serve as an antiviral target.

## Abstract (precis)

Outbreaks of infections with EV-A71, which causes hand, foot, and mouth disease, severe neurological disorders, and even death, have been repeatedly reported worldwide in recent decades and are a great public health problem for which no approved treatments are available. We show that Hsp27, a heat shock protein, supports EV-A71 infection in two distinct ways to promote viral IRES-dependent translation. A small-molecule Hsp27 inhibitor isolated from a traditional Chinese medicinal herb effectively reduces virus yields. Together, our findings demonstrate that Hsp27 plays an important role in EV-A71 infection and may serve as an antiviral target.

## INTRODUCTION

Hand, food, and mouth disease (HFMD), mostly caused by enterovirus A71 (EV-A71) and coxsackievirus A16 (CV-A16) infections, has become a global challenge for public health (1, 2). HFMD is usually a self-limited disease with mild clinical manifestations. However, in some cases of EV-A71 infection, it causes serious neurological complications and even death in children, especially those under 5 years old. These complications include poliomyelitis-like acute flaccid paralysis, aseptic meningitis, brain stem encephalitis, and pulmonary edema (3, 4). EV-A71 was first isolated in 1969 in California, and in 1973 it was recognized as a pathogen causing HFMD in Japan (5). Sporadic and epidemic outbreaks of EV-A71 have been repeatedly reported in recent decades, especially in the Asia-Pacific region (6). Although much work has been done to develop drugs for treating EV-A71 infections and several potential antiviral agents, including rupintrivir (AG7088), DTriP-22, kaempferol, NITD008, rocaglamide (Roc-A), oblongifolin M, and Ver-155008, have been identified (1, 7–11), there are, unfortunately, no available prophylactic or therapeutic agents active against EV-A71 infection.

EV-A71 is a nonenveloped, positive-sense single-stranded RNA (+ssRNA) virus and is a member of the *Enterovirus* genus in the *Picornaviridae* family (2, 12). The EV-A71 genome is about 7.4 kb in length and is composed of a single open reading frame (ORF) flanked by two untranslated regions (UTRs; the 5′ UTR and the 3′ UTR). The 5′ UTR contains a type I internal ribosomal entry site (IRES). After initial infection, EV-A71 binds to host receptors (SCARB2, PSGL1) to enter host cells and releases its viral genome RNA into the cytoplasm. Then, a single polyprotein is synthesized through IRES-mediated translation. This polyprotein is proteolytically cleaved into various structural viral proteins (VP1 to VP4) and nonstructural viral proteins (2A to 2C, 3A to 3D) by the virus proteases 2A^pro^ and 3C^pro^ (6, 13–17). Because of the small genome with a limited coding capacity, EV-A71 utilizes host factors to support viral protein translation and genome replication. IRES *trans*-acting factors (ITAFs) are required to recruit the ribosome and bind IRES for initiating translation in cooperation with some canonical translation initiation factors (18, 19). In the past, a number of ITAFs have been identified, including FBP1, FBP2, hnRNP A1, hnRNP K, AUF1/hnRNP D, Sam68, HuR, and Ago2 (20, 21). 2A^pro^ and 3C^pro^ cleave eukaryotic initiation factor 4G (eIF4G) and poly(A) binding protein (PABP), which are essential for the cap-dependent translation. The cleavages lead to the shutoff of some host mRNA translation but favor viral protein synthesis (22–25). 2A^pro^ also cleaves FBP1, and the cleavage product, FBP1_1–371_, cooperates with the full-length FBP1 to positively regulate IRES-dependent translation (19). The NLRP3 inflammasome protects mice against EV-A71 infection, while it is cleaved by 2A^pro^ and 3C^pro^ to facilitate virus infection (26). The underlying mechanism of host-virus interactions is not yet well understood.

Although cell proteins play an important role in the virus life cycle, our knowledge of what and how host proteins participate in and regulate EV-A71 infections is still very limited. In the present study, we performed a proteomic analysis to identify host factors responding to EV-A71 replication. We show that Hsp27 is markedly elevated during EV-A71 replication. By using gain- and loss-of-function studies, we dissect the importance of Hsp27 in EV-A71 infection and show that an Hsp27 inhibitor exhibits potent antiviral effects.

## RESULTS

### Proteomics analysis of cellular protein profiles upon EV-A71 infection.

**FIG 1:**
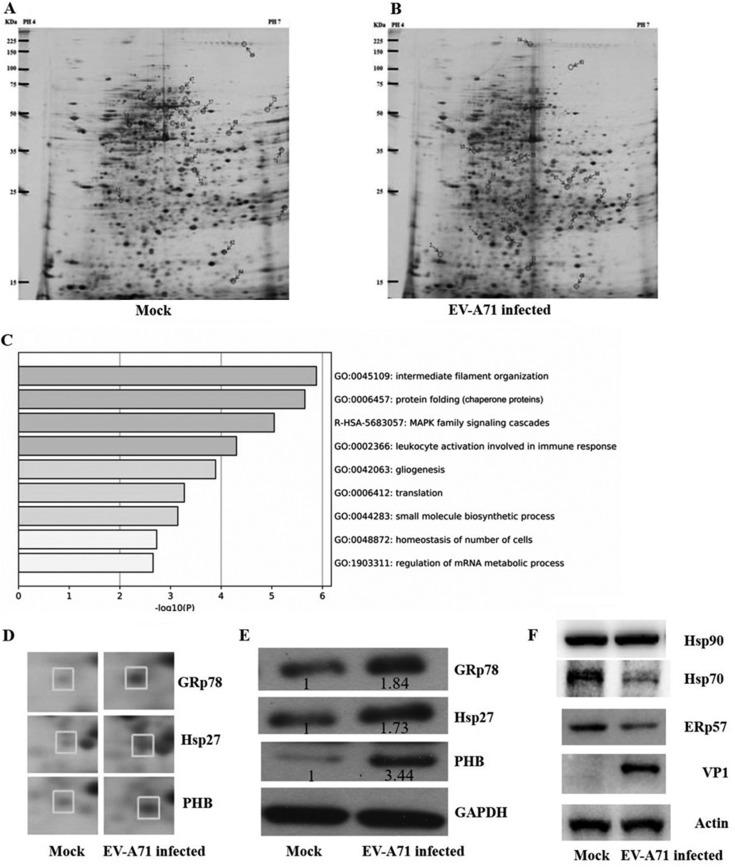
Protein profile in EV-A71-infected and noninfected RD cells. (A and B) Representative silver-stained 2-DE maps of proteins from EV-A71-mock-infected (noninfected) (A) and EV-A71-infected (B) RD cells (*n* = 3). Spots that were successfully identified by MALDI-TOF-MS and MS/MS scanning are marked with numbers. (C) Genes with significant differences in expression between infected and uninfected RD cells were subjected to GO analyses. The log_10_ values of the *P* values are indicated by bar plots. MAPK, mitogen-activated protein kinase. (D) Enlarged sections of the 2-DE maps showing different expressions of spot 10 (GRp78), spot 49 (Hsp27), and spot 7 (PHB) between EV-A71-infected and noninfected (mock-infected) RD cells. (E and F) RD cells were either mock infected or infected with EV-A71 at an MOI of 10 for 6 h. A Western blot assay was used to detect the expression of GRp78, PHB, and Hsp27 (the relative fold change of protein expression is indicated) (E) and other stress response proteins, ERp57, Hsp70, and Hsp90 (F).

The rhabdomyosarcoma (RD) cell line, which has been widely used to study the infection mechanism of enteroviruses, was used for proteomic analysis in this study. Our previous work on the kinetics of EV-A71 infection showed that both viral RNA synthesis and protein translation are highly active during the time phase of 6 to 9 h postinfection (p.i.) when cells are infected at a multiplicity of infection (MOI) of 1 or 10. Besides, progeny virions are also increasingly packaged and released over the same period (27). To identify host factors responding to early virus replication, proteins were extracted and applied for proteomics analysis at 6 h p.i. The extracts from RD cells with or without EV-A71 infection were analyzed by two-dimensional gel electrophoresis (2-DE). After comparing replicate 2-DE gels (*n* = 3) from infected and noninfected cells, a total of 73 protein spots were found to be consistently altered (>2-fold changes, *P* < 0.05), and of these, 36 displayed an increased expression level and 37 displayed a reduced expression level in the infected cells (Fig. 1A and B). Among these, 37 spots were picked for matrix-assisted laser desorption ionization–time of flight (MALDI-TOF) mass spectrometry (MS) and tandem MS (MS/MS) analysis. After removing the duplicates (e.g., precursor proteins), 24 proteins were eventually identified. Half of the proteins were upregulated in EV-A71-infected cells. The characteristics of all identified proteins, including the protein name, NCBI protein accession number, fold change in expression, protein score, number of mass values matched/searched, theoretical molecular mass/pI, and expected value, are listed in Table 1. In order to characterize the cellular response during EV-A71 infection, the Gene Ontology (GO) database was used to screen the functions of these significantly altered proteins (Fig. 1C). We found that proteins involved in cell stress responses changed the most upon EV-A71 infection.

**TABLE 1:** Proteins identified by 2-DE and MS analysis to be differentially expressed between EV-A71-infected and noninfected RD cells

Spot no.^*a*^	Gene name	Function	NCBI protein accession no.	Theoretical *M*_r_/pI	Score	Fold change in expression^*b*^	Peptide count (no.)	Sequence coverage (%)
6	KRT9	Cytoskeleton	P35527	62.3/5.14	80	3.7	13	31
7	**PHB**^*c*^	Cell stress response	P35232	29.8/5.57	83	5	8	30
10	**GRp78**	Cell stress response	P11021	72.4/5.07	73	3.5	12	20
16	VCP	Cellular metabolism	P55072	90/5.14	131	−3.7	25	32
18	VIM	Cytoskeleton	P08670	53.2/5.06	227	5.5	31	60
26	ZNF836	Transcriptional factors	Q6ZNA1	111/9.39	71	−3.1	11	16
30	PSMC3	Cellular metabolism	P17980	49.5/5.13	74	−3.2	13	35
33	SIPA1L1	Unknown function	O43166	201.1/8.4	69	10	19	14
37	FDFT1	Cellular metabolism	P37268	48.6/6.1	121	3.1	10	34
29	PSMC4	Cellular metabolism	P43686	47.5/5.09	128	−3.3	19	39
44	TUBB	Cytoskeleton	P07437	50.1/4.78	79	−4.5	11	34
45	DES	Cytoskeleton	P17661	53.6/5.21	281	−7	33	72
46	HNRNPK	Gene transcription	P61978	51.2/5.39	117	−3.3	14	29
47	NDUFS1	Cellular metabolism	P28331	80.4/5.89	78	−3.2	13	21
49	**Hsp27**	Cell stress response	P04792	22.8/5.98	80	5.6	7	37
51	EIF3I	Gene translation	Q13347	36.9/5.38	122	−4.6	13	49
52	PPA1	Cellular metabolism	Q15181	33.1/5.54	71	−4	11	49
57	**ERp57**	Cell stress response	P30101	57.1/5.98	98	−3.2	14	31
59	**HSPA9**	Cell stress response	P38646	73.9/5.87	67	−3.2	10	18
71	PKM2	Cellular metabolism	P14618	58.5/7.96	87	−3.7	13	29
72	PHGDH	Cellular metabolism	O43175	57.4/6.29	77	−5	12	23
80	ANXA1	Transport protein	P04083	38.9/5.1	73	3.8	11	22
91	SPTA1	Cytoskeleton	P02549	28.1/4.95	76	3.5	30	25
92	EF2	Gene translation	P13639	96.2/6.41	93	5	20	16

a Spot numbers are shown in Fig. 1.

b The spot intensities were quantified using PDQuest software (Bio-Rad). The average fold change in the spot intensity for each protein (for EV-A71-infected versus noninfected cells) was calculated from three independent experiments; a minus sign indicates a decrease.

c Stress response proteins are highlighted in bold.

### Protein validation by Western blot analysis.

To validate the results from the proteomics studies, we carried out a Western blot analysis using the extracts of RD cells that were either mock or EV-A71 infected at an MOI of 10 for 6 h. Three altered chaperone proteins (GRp78, PHB, and Hsp27) associated with the cell stress response were selected for use in the validation. Consistent with the observations from the 2-DE analysis, all three proteins were upregulated (Fig. 1E). To address if the increase in the amount of Hsp27 is a general response or a kind of specific chaperone response to EV-A71 infection, we also detected the expression level of other chaperone proteins. We observed that the levels of ERp57 and Hsp70 even decreased after virus infection, while the expression of Hsp90 did not show any obvious change (Fig. 1F).

### Upregulation of Hsp27 expression in EV-A71-infected cells.

**FIG 2:**
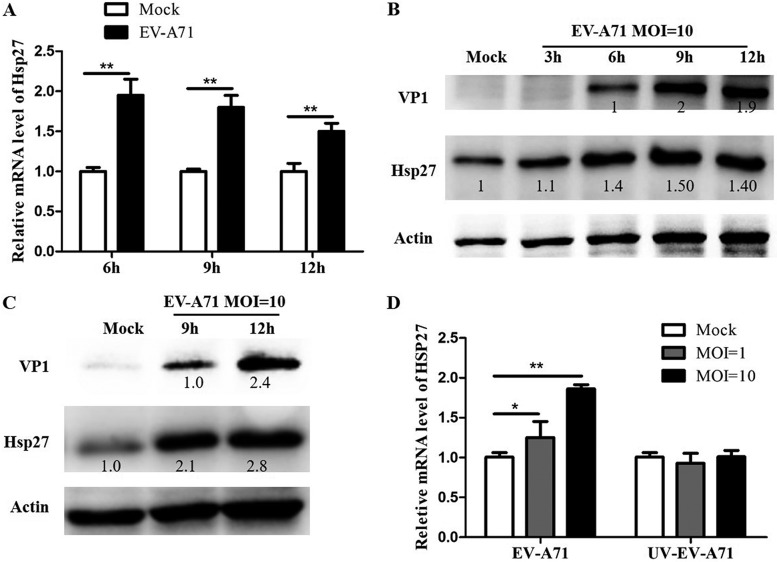
Upregulation of Hsp27 expression in EV-A71-infected RD cells. (A) RD cells were infected with or without EV-A71 at an MOI of 10. The expression level of Hsp27 was determined by RT-qPCR. (B) RD cells were infected with or without EV-A71 at an MOI of 10 for 3 h, 6 h, 9 h, and 12 h. The Hsp27 expression level was confirmed by Western blot assays. The density of each band was scanned and first normalized to that of actin. The value for the target protein band was set at 1 in the mock-infected control. (C) HeLa cells were infected with EV-A71 at an MOI of 10 for 9 h or 12 h. The expression levels of Hsp27 and viral protein VP1 were determined by a Western blot assay. (D) RD cells were infected with infectious EV-A71 or UV-inactivated EV-A71 at an MOI of 1 or 10 for 12 h. The expression level of Hsp27 was determined by RT-qPCR. GAPDH was used as the internal control. The experiments were conducted in triplicate and repeated at least three times. Statistical analyses were carried out using Student's *t* test. *, *P* < 0.05; **, *P* < 0.01. Data are expressed as the mean ± SD from three experiments.

To reveal the dynamic changes of Hsp27 during EV-A71 infection, we analyzed the mRNA and protein levels of Hsp27. Total RNA as well as proteins was extracted from EV-A71-infected cells and measured at different time points. As shown in Fig. 2A, the mRNA level of Hsp27 significantly increased by 100% at 6 h p.i. and 80% at 9 h p.i. Similarly, the protein levels were also significantly elevated over 40% to 50% at 6, 9, and 12 h p.i., when viral protein VP1 reached a relatively high level (Fig. 2B). These results were further confirmed in HeLa cells (Fig. 2C), indicating that EV-A71 infection stimulated Hsp27 expression. Moreover, the new synthesis of viral protein was first detected at 6 h p.i., matching the time point with the significant increase of Hsp27 (Fig. 2B). We postulated that the increased Hsp27 level may depend on EV-A71 replication. To demonstrate this hypothesis, we treated RD cells with UV-inactivated EV-A71 by exposing virions to light from a UV lamp as previously described (28). Unlike infectious EV-A71, UV-inactivated EV-A71 was not able to increase Hsp27 expression (Fig. 2D). This result suggests that EV-A71 replication is indispensable for the upregulation of Hsp27.

### Knockout of Hsp27 reduced EV-A71 replication.

**FIG 3:**
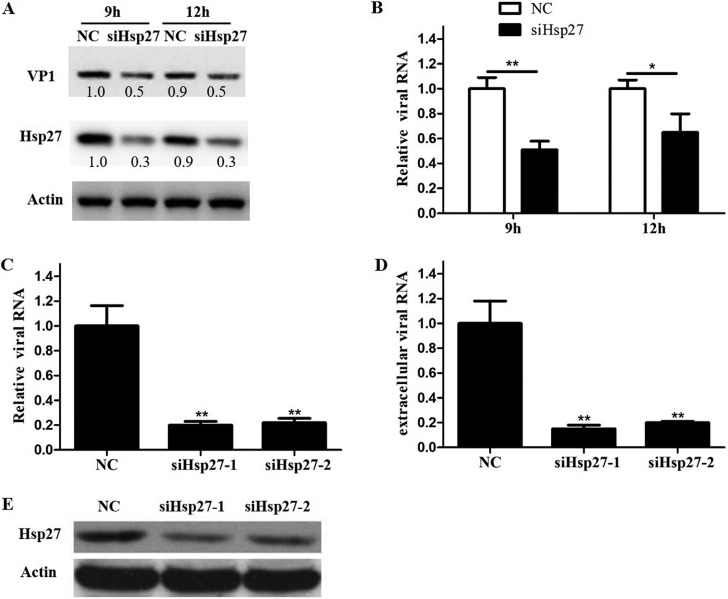
Inhibition of EV-A71 replication by knockdown of Hsp27 in HeLa and RD cells. (A and B) HeLa cells were transfected with negative-control siRNA (NC) or Hsp27 siRNA (siHsp27) for 48 h, and then the cells were infected with EV-A71 and the intracellular viral protein level of VP1 (A) and the viral RNA level (B) were determined by Western blotting and RT-qPCR, respectively. (C to E) RD cells were transfected with negative-control or Hsp27 siRNA for 48 h, and then the cells were infected with EV-A71. (C and D) The EV-A71 mRNA levels in both the cytoplasm (C) and the supernatant (D) were evaluated by RT-qPCR. (E) The Hsp27 level was confirmed by Western blotting at 48 h posttransfection. GAPDH was used as the internal control. Statistical analyses were carried out using Student's *t* test. *, *P* < 0.05; **, *P* < 0.01. Data are expressed as the mean ± SD.

**FIG 4:**
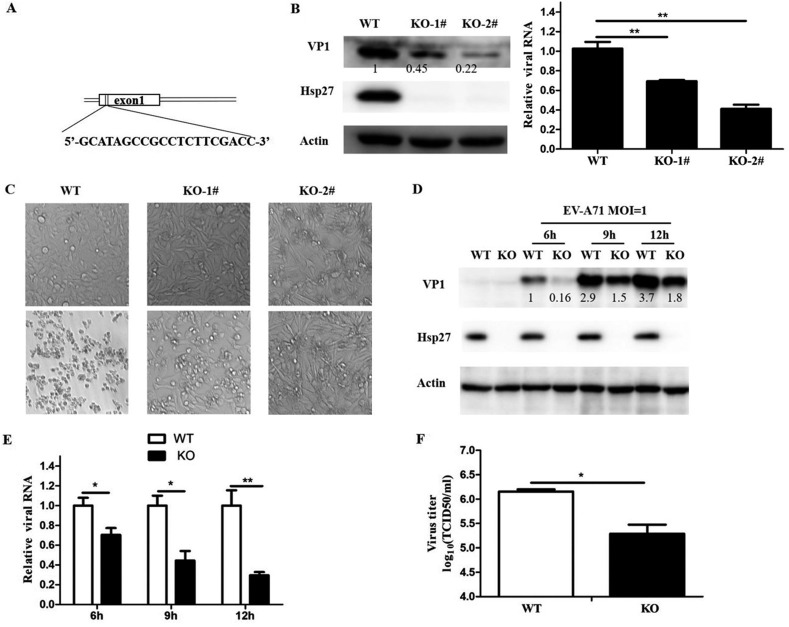
Inhibition of EV-A71 replication by knockout of Hsp27. (A) The designed target site in exon 1 of Hsp27 by CRISPR/Cas9. (B) Hsp27 was knocked out in RD cells, and the cells were infected with EV-A71 at an MOI of 1 for 6 h. Viral protein and viral RNA levels were determined by Western blot assay and RT-qPCR, respectively. (C) RD wild-type (WT), Hsp27-KO clone 1 (KO-1), and Hsp27-KO clone 2 (KO-2) cells were infected with EV-A71 at an MOI of 0.01. Photos were taken at 48 h postinfection (p.i.). The wild-type and Hsp27-KO cells were infected with EV-A71 at an MOI of 1 for 6, 9, and 12 h. (D) The viral protein level was determined by Western blot assay. (E) The intracellular viral RNA level was examined by RT-qPCR. (F) The virus titer in the supernatant was determined by TCID_50_ assay. GAPDH was used as the internal control. Statistical analyses were carried out using Student's *t* test. *, *P* < 0.05; **, *P* < 0.01. Data are expressed as the mean ± SD.

**FIG 5:**
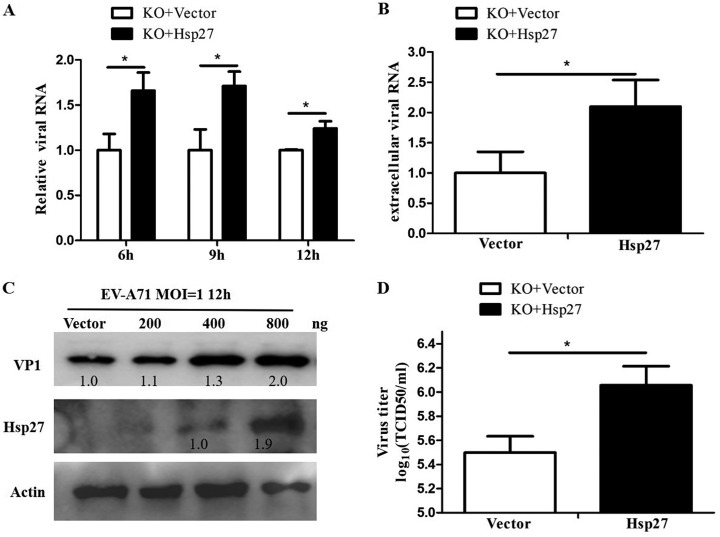
Promotion of EV-A71 replication by restored Hsp27 expression in Hsp27-KO cells. Hsp27-KO cells were transfected with the empty vector and plasmid pHsp27, and at 48 h posttransfection, the cells were infected with EV-A71. The intracellular (A) and extracellular (B) viral RNA levels, the viral protein level (C), and the viral titer in the supernatant (D) were determined by RT-qPCR, Western blot assay, and TCID_50_ assay, respectively. GAPDH was used as the internal control. Statistical analyses were carried out using Student's *t* test. *, *P* < 0.05. Data are expressed as the mean ± SD.

The elevated Hsp27 response to active EV-A71 infection only suggested that Hsp27 may play a crucial role in viral reproduction. To assess the potential function of Hsp27 in the process of EV-A71 infection, we first used small interfering RNA (siRNA) to knock down Hsp27 in RD and HeLa cells. It was shown that knockdown of Hsp27 significantly inhibited viral replication (Fig. 3). To further confirm our findings, we created Hsp27 knockout (Hsp27-KO) cells using a CRISPR/Cas9-mediated deletion strategy. We chose exon 1 of Hsp27 (Fig. 4A) as the target site to generate knockout cell lines. RD cells were transfected with a human Hsp27 CRISPR/Cas9 knockout plasmid and then selected with puromycin. The selected colony was further determined by Western blot assays. As shown in Fig. 4B, Hsp27 was completely knocked out in RD cells. Two Hsp27-KO clones were used to test the function of Hsp27 in EV-A71 replication. Compared with the level in wild-type cells, the viral RNA level decreased by 31% and 60% in clones 1 and 2, respectively, while VP1 protein levels were reduced by 55% in clone 1 and 78% in clone 2. The cytopathic effects (CPE) caused by EV-A71 infection were also protected by the knockout of Hsp27 (Fig. 4C). Because clone 2 showed a much stronger effect for limiting viral replication, this clone was chosen for the following experiments. The role of Hsp27 in EV-A71 infection was further confirmed in a time-dependent manner. The wild-type cells as well as Hsp27-KO cells were infected with EV-A71 at an MOI of 1 for 6, 9, and 12 h. Both viral protein and RNA levels were determined by Western blot and real-time quantitative PCR (RT-qPCR) assays, respectively. As shown in Fig. 4D, viral protein VP1 could not be detected in the Hsp27-KO cells at the time point of 6 h, whereas it was already obvious at this time point in the wild-type cells. Although it was able to be detected later on, the VP1 level markedly decreased by over 50% in the Hsp27-KO cells at 9 and 12 h p.i. Consistent with the reduction of VP1 levels, the intracellular viral RNA levels also decreased in the Hsp27-KO cells (Fig. 4E). Surprisingly, the virus yield was 8-fold more in the culture medium of wild-type cells than in that of Hsp27-KO cells (Fig. 4F). More importantly, Hsp27 markedly enhanced the viral RNA and protein levels when we restored Hsp27 expression in the Hsp27-KO cells (Fig. 5A to C). The virions in the supernatant also increased about 3.6-fold after restoration of Hsp27 in the knockout cells (Fig. 5D).

### Ectopic expression of Hsp27 promoted EV-A71 reproduction.

**FIG 6:**
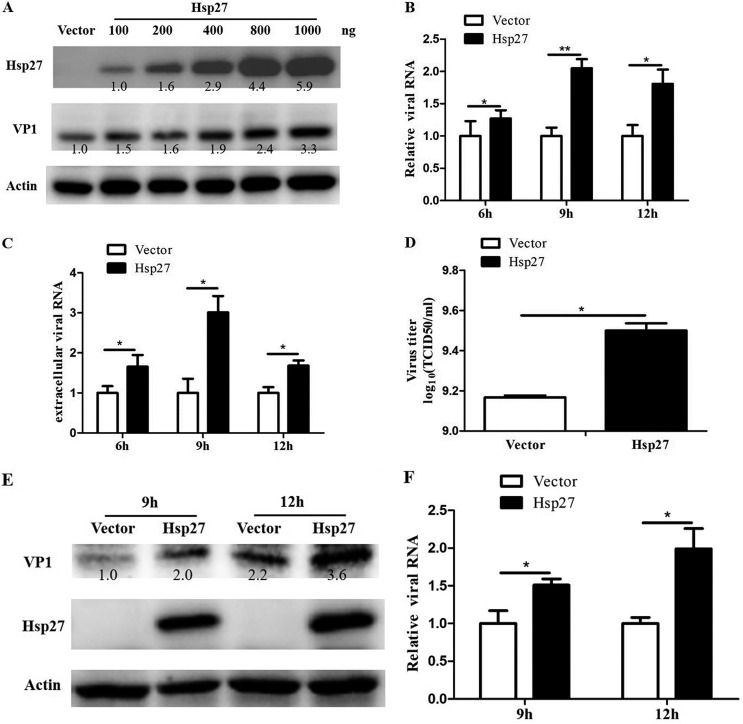
Promotion of EV-A71 infection by ectopic expression of Hsp27. (A to D) HEK 293T cells were transfected with an Hsp27 expression plasmid for 48 h. (A) The cells were then infected with EV-A71 at an MOI of 1 for 12 h. The expression levels of Hsp27 and viral protein VP1 were detected by Western blot assay. (B and C) The cells were infected with EV-A71 at an MOI of 1 for 6 h, 9 h, and 12 h, and the intracellular (B) and extracellular (C) viral RNA levels were examined by RT-qPCR. (D) The virus titer in the supernatant was determined by TCID_50_ assay. (E and F) HeLa cells were transfected with the empty vector or plasmid pHsp27. At 48 h posttransfection, the cells were infected with EV-A71 at an MOI of 1 for 9 h and 12 h, and the level of viral protein VP1 and the viral RNA level were detected by Western blot assay and RT-qPCR, respectively. GAPDH was used as the internal control. Statistical analyses were carried out using Student's *t* test. *, *P* < 0.05. Data are expressed as the mean ± SD.

To further validate the functions of Hsp27 in EV-A71 infection, we conducted gain-of-function studies by transfecting HEK 293T cells with an Hsp27-expressing plasmid. At 48 h posttransfection, the cells were infected with EV-A71 at an MOI of 1 for 12 h. The intracellular protein levels of both Hsp27 and viral VP1 were examined by Western blot assays. As shown in Fig. 6A, both Hsp27 and viral VP1 protein levels significantly increased in a dose-dependent manner. More importantly, the increase of VP1 levels positively corrected with the levels of Hsp27. In correspondence with the increase of VP1 and Hsp27 levels, both the intracellular viral RNA and secreted virion RNA levels were almost doubled or even tripled at 6 h and 12 h p.i. when the cells were transfected with 400 ng of Hsp27-expressing plasmid (Fig. 6B and C). The viral titer in the supernatant also markedly increased 3- to 4-fold after ectopic expression of Hsp27 (Fig. 6D). Similar results were also obtained from HeLa cells (Fig. 6E and F).

### Hsp27 upregulates EV-A71 IRES activity.

**FIG 7:**
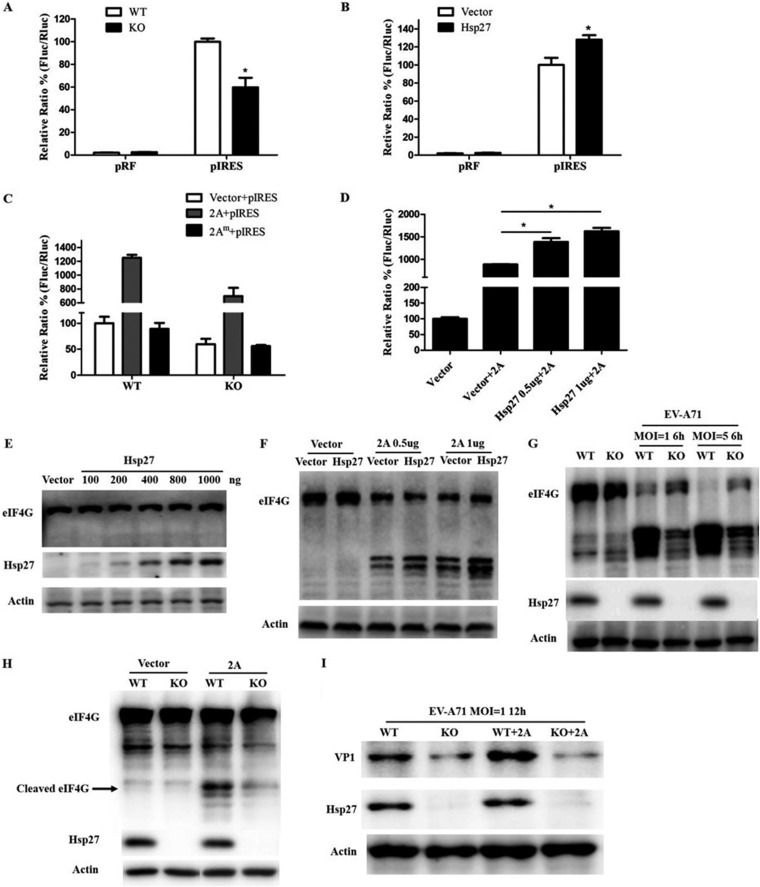
Upregulation of viral IRES activity by Hsp27. (A) Wild-type and Hsp27-KO RD cells were transfected with the pIRES reporter plasmid. The luciferase activity was determined at 48 h posttransfection as described in the Materials and Methods section. (B) HEK 293T cells were cotransfected with pHsp27 and the pIRES reporter plasmid for 48 h, and luciferase activity was then measured. (C) Wild-type and Hsp27-KO RD cells were cotransfected with the control or 2A^pro^-expressing plasmid and the pIRES reporter plasmid for 48 h, and then the luciferase activity was measured. (D) HEK 293T cells were cotransfected with pHsp27, the pIRES reporter plasmid and a 2A^pro^-expressing plasmid, or the empty vector for 48 h, and then the luciferase activity was measured. (E) HEK 293T cells were transfected with pHsp27 for 48 h, and the expression levels of eIF4G and Hsp27 were determined by Western blot assay. (F) HEK 293T cells were cotransfected with pHsp27 and the 2A^pro^-expressing plasmid or the empty vector for 48 h, and the cleaved eIF4G was determined by Western blot assay. (G) Wild-type and Hsp27-KO RD cells were infected with EV-A71 at an MOI of 1 or 5 for 6 h, and the cleaved eIF4G was determined by Western blot assay. (H) Wild-type and Hsp27-KO RD cell lysates were collected and incubated with recombinant 2A^pro^ at 37°C for 4 h in a cleavage buffer, and the cleaved eIF4G was determined by Western blot assay. (I) Wild-type and Hsp27-KO RD cells were transfected with a 2A^pro^-expressing plasmid or the empty vector for 24 h, and then the cells were infected with EV7-A71 at an MOI of 1 for 12 h and the levels of viral VP1 and the Hsp27 protein were determined by Western blot assay. Statistical analyses were carried out using Student's *t* test. *, *P* < 0.05. Data are expressed as the mean ± SD.

To determine whether Hsp27 has an effect on EV-A71 IRES activity, we transfected a dicistronic reporter plasmid (11) into wild-type and Hsp27-KO RD cells. After culture for 48 h, *Renilla* luciferase (RLuc) and firefly luciferase (FLuc) activities were determined with a dual-luciferase assay kit. The normalized ratio of Fluc to Rluc presented the relative IRES activity. As shown in Fig. 7A, knockout of Hsp27 led to a decrease in EV-A71 IRES activity of 40%. On the other hand, ectopic expression of Hsp27 resulted in about a 30% increase in IRES activity (Fig. 7B). After we transfected a 2A^pro^ expression plasmid (29) into RD cells, the IRES activity was stimulated more than 12-fold, while the protease-deficient mutant showed no effect on the stimulation of IRES activity. Strikingly, the baseline IRES activity decreased over 40% in the Hsp27-KO cells. Furthermore, the IRES activity was also reduced about 40% in the Hsp27-KO cells from that in wild-type cells when 2A^pro^ was ectopically expressed (Fig. 7C). Consistent results were also obtained from experiments in HEK 293T cells. Ectopic expression of Hsp27 increased the 2A^pro^-mediated IRES activity in a dose-dependent manner (Fig. 7D). To further investigate the effects of Hsp27 on 2A^pro^ protease activity, we first detected whether Hsp27 upregulated the expression level of eIF4G. We showed that the eIF4G level was not disturbed by ectopically expressed Hsp27 (Fig. 7E). We then checked if Hsp27 affected eIF4G cleavage, a marker of 2A^pro^ protease activity. As shown in Fig. 7F, surprisingly, Hsp27 strongly enhanced 2A^pro^ protease activity no matter whether more or less 2A^pro^-expressing plasmid was used in these experiments. In the Hsp27-KO cells, the cleavage of eIF4G was significantly decreased to nearly the basal level at 6 h when the cells were infected with EV-A71 at an MOI of 1 (Fig. 7G). Almost the same results were observed when recombinant 2A^pro^ expressed in Escherichia coli was incubated *in vitro* with the lysates from wild-type and Hsp27-KO cells at 37°C for 4 h (Fig. 7H). Finally, we showed that ectopic expression of 2A^pro^ promoted viral VP1 expression in the wild-type RD cells (Fig. 7I, third lane versus first lane), whereas the knockout of Hsp27 almost diminished viral VP1 expression with or without the promotion by 2A^pro^ (Fig. 7I, fourth lane versus third lane and first lane).

### Knockout of Hsp27 inhibited EV-A71-induced hnRNP A1 translocation.

**FIG 8:**
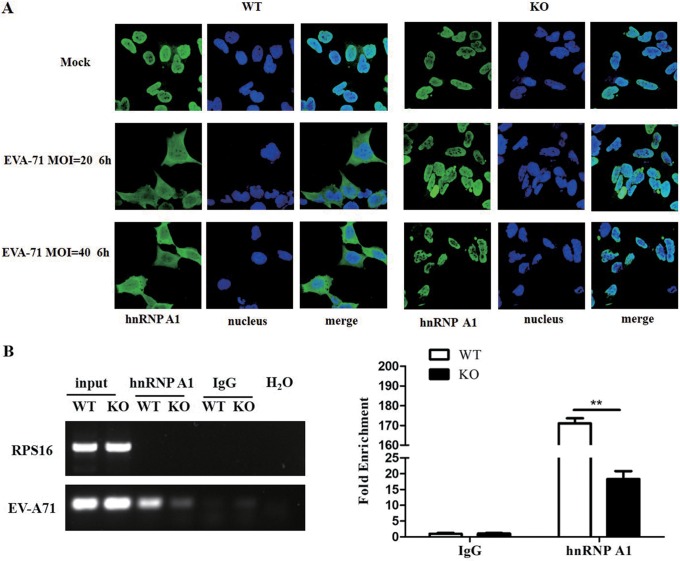
Inhibition of EV-A71-induced hnRNP A1 translocation by knockout of Hsp27. (A) Wild-type and Hsp27-KO cells RD were infected with EV-A71 at an MOI of 20 or 40 for 6 h. The cells were fixed, and the subcellular localization of hnRNP A1 was investigated by indirect immunofluorescence assay. (B) Wild-type and Hsp27-KO RD cells were infected with EV-A71 at an MOI of 10 for 6 h. Cell extracts were used for RNA immunoprecipitation assay with anti-hnRNP A1 antibody or normal anti-IgG. After washing and dissociation, the RNA was extracted using the TRIzol reagent and subjected to RT-PCR using primers that were specific to either the EV-A71 5′ UTR or ribosomal protein S16 (RPS16). Statistical analyses were carried out using Student's *t* test. **, *P* < 0.01. Data are expressed as the mean ± SD.

The hnRNPs A1 and FBP1 play critical roles in enhancing IRES-dependent translation (20). After EV-A71 infection, hnRNP A1 translocates from the nucleus to the cytoplasm and interacts with the EV-A71 5′ UTR (30). We postulated that Hsp27 may affect hnRNP A1 and FBP1 redistribution to regulate IRES activity. Wild-type and Hsp27-KO RD cells were infected with EV-A71 at an MOI of 20 or 40 for 6 h, and then we visualized the intracellular hnRNP A1 protein distribution via immunofluorescence staining. We showed that hnRNP A1 normally localized in the nucleus in the mock-infected cells (Fig. 8A, top) and translocated from the nucleus to the cytoplasm after EV-A71 infection (Fig. 8A, middle and bottom left). However, the translocation of hnRNP A1 was almost completely blocked in Hsp27-KO cells (Fig. 8A, middle and bottom right). We further conducted an RNA immunoprecipitation (RIP) assay to dissect the binding activity of hnRNP A1 to the viral 5′ UTR. As shown in Fig. 8B, knockout of Hsp27 resulted in a significant reduction of viral RNA binding with hnRNP A1.

**FIG 9:**
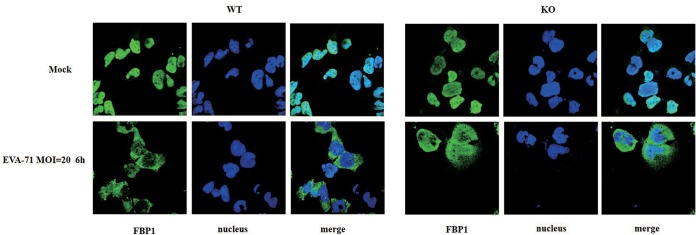
No effects on FBP1 translocation by knockout of Hsp27. Wild-type and Hsp27-KO RD cells were infected with EV-A71 at an MOI of 20 for 6 h. The cells were fixed, and the subcellular localization of FBP1 was investigated by indirect immunofluorescence assay.

Surprisingly, Hsp27 knockout did not block FBP1 translocation after EV-A71 infection (Fig. 9), suggesting that Hsp27 differentially regulates only a portion of the RNA binding proteins’ redistribution to the cytosol.

### The Hsp27 inhibitor TDP suppressed EV-A71 infection.

**FIG 10:**
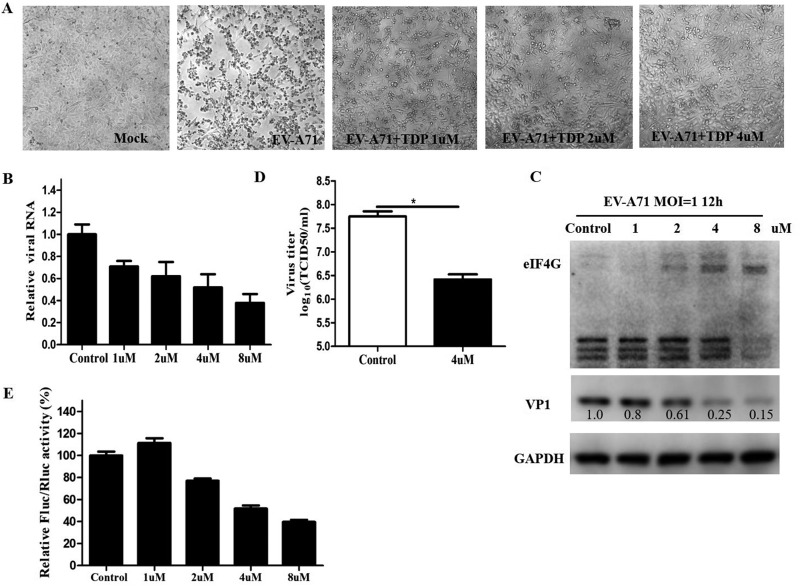
Suppression of EV-A71 infection by a small-molecule inhibitor of Hsp27. (A) RD cells were pretreated with TDP for 2 h at the indicated concentrations and then infected with EV-A71 at an MOI of 0.01. Photos were taken at 48 h postinfection (p.i.). RD cells were pretreated with TDP for 2 h at the indicated concentrations and then infected with EV-A71 at an MOI of 1 for 12 h. (B) The intracellular viral RNA level was detected by RT-qPCR. (C) The expression level of eIF4G and the level of viral protein VP1 were determined by Western blot assay. (D) The viral titer in the supernatant was determined by TCID_50_ assay. (E) The pIRES reporter plasmid was transfected into cells which had been pretreated with TDP for 2 h, and IRES activity was measured at 48 h posttransfection. Statistical analyses were carried out using Student's *t* test. *, *P* < 0.05; **, *P* < 0.01. Data are expressed as the mean ± SD.

1,3,5-Trihydroxy-13,13-dimethyl-2*H*-pyran [7,6-*b*] xanthone (TDP), a natural product which was isolated from the traditional Chinese medicinal herb Garcinia oblongifolia, has proved to be an inhibitor of Hsp27 (31). We hypothesized that TDP could inhibit EV-A71 replication. The antiviral effect of TDP was tested in RD cells by CPE assays. Cells were pretreated with TDP for 2 h and then infected with EV-A71 at an MOI of 0.01. At 48 h postinfection, a CPE was detected using a phase-contrast microscope. As shown in Fig. 10A, the cells became round and detached from the dishes after EV-A71 infection. TDP treatment protected the cells from EV-A71 infection and dramatically reduced the CPE in a dose-dependent manner. Consistent with the CPE reduction, TDP significantly inhibited EV-A71 replication, as shown by the largely reduced levels of intracellular viral RNA (Fig. 10B) and viral protein (Fig. 10C). Virus-induced eIF4G cleavage was also inhibited by TDP (Fig. 10C). Moreover, the viral yield in the supernatant significantly decreased after TDP treatment (Fig. 10D). Also, IRES activity was inhibited by TDP compared with the activity in the control group (Fig. 10E).

## DISCUSSION

In this study, we performed proteomics studies to reveal important host factors that respond to EV-A71 infection and further conducted comprehensive loss-of-function and gain-of-function studies to reveal the potential functions of Hsp27 in viral infection. We showed that Hsp27 was upregulated in response to virus replication. For the first time, we demonstrated that Hsp27 supports EV-A71 replication by enhancing IRES-driven viral protein translation. Hsp27 upregulates viral IRES activity through 2A^pro^-mediated cleavage of eIF4G and hnRNP A1 translocation from the nucleus to the cytoplasm. More importantly, an Hsp27 inhibitor, TDP, a compound isolated from a traditional Chinese medicinal herb, Garcinia oblongifolia, displayed potent antiviral activity against EV-A71 infection.

Although many efforts have been made, the host factors involved in facilitating or restricting EV-A71 infection are not fully defined. In this study, we identified by MALDI-TOF MS and MS/MS analysis 24 proteins differentially expressed after EV-A71 infection. Among them, a set of stress response proteins (including GRp78, PHB, and Hsp27) was significantly upregulated. It has been shown that GRp78 is an important responding factor that restricts viral activities upon EV-A71 infection (32). A recent proteomics study showed that PHB plays a crucial role in facilitating viral infection (9). The consistency of our results with other reports demonstrates the high quality of our study and suggests an important role of Hsp27 in EV-A71 infections, which has not been yet addressed.

Hsp27 belongs to the large heat shock protein (HSP) family, which is conserved and ubiquitously expressed in most organisms from prokaryotes to eukaryotes. HSPs act as molecular chaperones to protect the cells and inhibit protein aggregation in response to heat shock or other cellular stresses, such as pathogen invasion (33–35). Hsp90 has been reported to be involved in various virus infections, including hepatitis B virus (HBV), hepatitis C virus (HCV), HIV, Epstein-Barr virus (EBV), and Sendai virus infections (36). Besides EV-A71, GRp78 also plays a crucial role in many other virus infections, including those caused by HBV, dengue virus (DENV), Japanese encephalitis virus (JEV), and West Nile virus (WNV) (37, 38). It has been reported that Hsp27 is phosphorylated upon HSV-1 infection and that depletion of Hsp27 reduces herpes simplex virus 1 production (39). Treatment with the p38 mitogen-activated protein kinase inhibitor SB203580 reduces the liver injury induced by DENV infection, partially by suppressing the phosphorylation of Hsp27 (40). In the case of HBV infection, Hsp27 acts as an antiviral factor by inducing type I interferon and downstream antiviral effectors (ISG15, OAS1, OAS3, protein kinase R, and EIF2a) (41). These studies demonstrate that Hsp27 plays different roles in different viruses in given cells. In the present study, we showed that Hsp27 expression was significantly unregulated in the course of viral replication (Fig. 2A to C), whereas UV-inactivated replication-deficient EV-A71 failed to stimulate Hsp27 expression (Fig. 2D). Our results demonstrate that Hsp27 responds to EV-A71 replication instead of virus entry. It is reasonable to postulate that the Hsp27 response must have exhibited a great impact on virus activities, either promoting or restricting EV-A71 replication. Clearly, our data demonstrate that the knockout of Hsp27 significantly reduces EV-A71 replication (Fig. 4). In contrast, ectopic expression of Hsp27 promotes the replication of EV-A71 (Fig. 6).

After entry and uncoating, the viral +ssRNA of enterovirus is immediately translated into a polypeptide through 5′ IRES-mediated viral protein translation, a key step to initiate viral replication (13, 18). It has been shown that Hsc70, another HSP family member, upregulates EV-A71 IRES activity to facilitate EV-A71 replication (42), and Hsp70 interacts with NS5A to regulate HCV IRES-mediated translation and support HCV replication (43). Our results revealed that the depletion of Hsp27 markedly reduces viral protein expression. To address whether Hsp27 participates in viral translation by regulating viral IRES activity, we performed reporter assays. We showed that the knockout of Hsp27 significantly reduced viral IRES activities (Fig. 7A), while ectopic expression of Hsp27 increased viral IRES activities (Fig. 7B), indicating an important function of Hsp27 in regulating viral IRES activities. It is well-known that the viral protein 2A^pro^ cleaves the cellular translation initiation factor eIF4G to shut off some host cap-dependent translation and to promote IRES-mediated viral RNA translation (25). Viral protein 2A^pro^ stimulates IRES-mediated translation, which depends on enzymatic activity (44, 45). In our study, we showed that the IRES activity was not affected by the enzyme activity-deficient 2A^pro^ mutant either in wild-type cells or in Hsp27-KO cells (Fig. 7C). The viral IRES activity significantly increased over 12-fold in wild-type cells; however, the increase in IRES activity by 2A^pro^ was much less in Hsp27-KO cells (Fig. 7C). Furthermore, the 2A^pro^-enhanced IRES activity positively correlated with the dose of ectopically expressed Hsp27 (Fig. 7D). Although it did not affect the expression level of eIF4G (Fig. 7E), Hsp27 significantly enhanced the 2A^pro^-mediated cleavage of eIF4G in a dose-dependent manner (Fig. 7F). Consistently, knockout of Hsp27 obviously blocked eIF4G cleavage no matter whether the cells were infected by EV-A71 or incubated with recombinant 2A^pro^ (Fig. 7G and H). The production of viral protein was strongly upregulated by 2A^pro^, and this positive regulation was abolished in Hsp27-KO cells (Fig. 7I). A recent study showed that Hsp25, the murine homologue of human Hsp27, is able to interact with eIF4G and incorporate into the eIF4F complex (46). Moreover, it has been demonstrated that the eIF4F complex is a preferred substrate for cleavage by 2A^pro^ (47). Taken together, it is reasonable that EV-A71 takes an efficient way to stimulate Hsp27 expression for promoting viral protein translation through enhancing eIF4G cleavage. It would not be surprising if our findings may also be applied to other positive-stranded RNA viruses containing IRES sequences, such as HCV and coxsackievirus.

Besides modulating eIF4G cleavage, it is also possible that Hsp27 takes other ways to regulate viral IRES activity. ITAFs are indispensable for IRES-dependent translation. As a member of the ITAFs, hnRNP A1 positively regulates IRES-dependent translation of EV-A71, Sindbis virus, HCV, and HIV (48, 49). It translocates from the nucleus to the cytoplasm after infection by EV-A71, HIV, or Sindbis virus (30, 50–53). In our study, we demonstrated that hnRNP A1 redistributes to the cytoplasm after EV-A71 infection, which is consistent with the findings of these previous studies. To our surprise, knockout of Hsp27 almost completely blocked hnRNP A1 translocation from the nucleus to the cytoplasm (Fig. 8A). More importantly, we further demonstrated by an RIP assay that the knockout of Hsp27 significantly reduced the interaction between hnRNP A1 and EV-A71 (Fig. 8B). There is no doubt that the reduced cytoplasmic relocation of hnRNP A1 also contributed to the decreased viral protein translation and replication seen in the Hsp27 knockout cells. However, Hsp27 knockout did not affect the translocation of FBP1 after EV-A71 infection (Fig. 9), indicating that the regulation of the RNA-binding protein cytosol redistribution is not general. TDP has been reported to be an inhibitor of Hsp27 (31); therefore, it is possible that TDP regulates EV-A71 infection. Our results demonstrate that TDP displays antiviral effects via the downregulation of IRES activity (Fig. 10), which indicates that TDP acts as a potential drug for treating EV-A71 infection.

Collectively, our study demonstrates that Hsp27 contributes to EV-A71 replication by positively regulating IRES activity. This provides a novel insight into the interaction between host factors and EV-A71, and Hsp27 could serve as a new target for the development of antiviral agents.

## MATERIALS AND METHODS

### Cells and virus.

Human muscle rhabdomyosarcoma (RD) cells (ATCC CCL-136) were maintained in Dulbecco’s modified Eagle’s medium (DMEM) containing 10% fetal bovine serum (FBS) with 100 U/ml penicillin and 100 μg/ml streptomycin. EV-A71 (SHZH98 strain, GenBank accession number AF302996) was propagated as previously described (29). The viral titer was determined by 50% tissue culture infective dose (TCID_50_) assays (29).

### Proteomics analysis.

Approximate 4 × 10^7^ RD cells were harvest at 6 h postinfection, and the mock-infected cells were set as the control. 2-DE was performed as described in a paper recently published by the M.-L. He lab (38). Briefly, cells were washed with phosphate-buffered saline (PBS) and lysed in lysis buffer (8 M urea, 2 M thiourea, 2% CHAPS {3-[(3-cholamidopropyl)-dimethylammonio]-1-propanesulfonate}, 1% NP-40, 2 mM tributylphosphine, 1× Roche protease inhibitor cocktail). The first-dimension isoelectric focusing (IEF) electrophoresis was performed in an Ettan IPGphor II IEF system (Amersham, USA) according to the manufacturer’s instructions. Protein samples (150 μg) were diluted with 250 μl rehydration solution (8 M urea, 2% CHAPS, 0.4% dithiothreitol [DTT], 0.5% immobilized pH gradient (IPG) buffer, 0.002% bromphenol blue) and applied to IPG strips (pH 4 to 7, linear; GE Healthcare). After IEF, the IPG strips were equilibrated in the balance solution (6 M urea, 2% SDS, 30% glycerol, 50 mmol/liter Tris-HCl [pH 6.8], 1% DTT) for 15 min and then transferred to another balance solution. The second-dimension electrophoresis was performed using 12.5% SDS-PAGE gels at 15 mA per gel for 30 min. Each experiment was performed in triplicate. After 2-DE, the gels were stained by a modified silver staining method. PDQuest two-dimensional analysis software (version 8.0; Bio-Rad) was applied for further analysis, including spot detection, background subtraction, spot matching, volume normalization, and quantitative intensity analysis. The intensity of each protein was quantified by calculation of the spot volume after normalization of the total density on each gel. Data were analyzed by Student's *t* test. The protein spots that were significantly differentially expressed (*P* < 0.05) according to at least a 2-fold increase/decrease (EV-A71-infected RD cells versus mock-treated control cells) in spot intensities were selected and subjected to identification by mass spectrometric (MS) analysis. Selected protein spots were excised from the preparative gels by using biopsy punches and purified with a Millipore ZipTip C_18_ column. Finally, a volume (1 μl) of the peptide samples was spotted on a stainless-steel plate for MS analysis with a 4700 proteomics analyzer (TOF/TOF; Applied Biosystems, USA).

### Database analysis.

Proteins were identified with peptide mass fingerprinting data by combining MS (peptide-mass-fingerprint approach) and MS/MS (*de novo* sequencing approach) analysis. In order to fit an ideal isotopic distribution to the experimental data, the Mascot Distiller program was applied for peak detection. Known contaminant ions corresponding to human keratin and trypsin autolysis peptides were first removed from the spectra before the database search. The raw data were submitted to the Mascot search engine (version 1.9.05; Matrix Science) and searched against the Swiss-Prot 20100723 database (518,415 sequences; 182,829,264 residues). Gene ontology (GO) clustering was performed using Metascape (http://metascape.org), and directed acyclic graphs on the same website were used to discover GO categories with enriched gene numbers (at least two genes in each categories [*P* < 0.01]).

### RNA interference.

Two small interfering RNAs (siRNAs) targeting human Hsp27 mRNA (siHsp27) were designed (GenBank accession number NM_001540) (for siHsp27-1, sense primer 5′-GGAUGGCGUGGUGGAGAUC-3′; for siHsp27-2, sense primer 5′-AGGAGUGGUCGCAGUGGUU-3′) and synthesized by GenePharma (Shanghai, China). Western blot assays were applied to measure the knockdown efficiency of the siRNAs. A nonspecific siRNA with no homology to the human genome was used as the negative control.

### Hsp27 knockout cells.

Human Hsp27 CRISPR/Cas9 knockout plasmids were purchased from GenScript. The target single guide RNA sequence was 5′-GCAUAGCCGCCUCUUCGACC-3′. RD cells were transient transfected with Hsp27 CRISPR/Cas9 knockout plasmids using the FuGENE HD transfection reagent. At 48 h after transfection, 2 μg/ml puromycin was added into the medium, incubation was continued for another 48 h, and then the surviving cells were reseeded at 1 cell per well into a 96-well plate. The expression level of Hsp27 in the expanded colonies was detected by Western blot analysis using anti-Hsp27 antibodies.

### Luciferase assays.

The reporter plasmids used in this study were described previously (11). HEK 293T cells were plated in a 24-well dish and cultured overnight. An Hsp27 expression plasmid or corresponding siRNA was transfected, and the cells were incubated for 24 h. The cells were then transfected with the pIRES or pRF reporter plasmid. At 2 days after the first-round transfection, cell extracts were prepared and assayed for *Renilla* luciferase (RLuc) and firefly luciferase (FLuc) activity in a Lumat LB9507 bioluminometer using a dual-luciferase reporter assay (Promega, USA) according to the manufacturer’s instructions.

### Western blot assay.

Cultured cells were washed with PBS, and the total cell lysates were collected by incubating the cells for 30 min on ice in a protein lysis buffer (50 mM Tris-HCl, pH 7.4, 150 mM NaCl, 1% Triton X-100, 0.1% SDS) supplemented with a protease inhibitor cocktail. After centrifugation at 4°C (12,000 × *g*) for 20 min, the supernatant was collected. Protein concentrations were measured using the Bradford method (Bio-Rad Laboratories, CA, USA), and 30 μg of protein was separated by 10% SDS-polyacrylamide gel electrophoresis and then transferred onto a polyvinylidene difluoride (PVDF) membrane (GE, MA, USA). The protein-containing membrane was blocked with 5% bovine serum albumin (BSA) in Tris-buffered saline–Tween 20 buffer (TBST; 20 mM Tris-HCl, pH 7.4, 150 mM NaCl, 0.1% Tween 20) at room temperature. The membrane was then incubated with antibodies against EV-A71 VP1 (1:2,000; Merck Millipore, MA, USA), Hsp27 (1:1,000; antibody sc-13132), eIF4G (1:1,000; antibody sc-11373), and β-actin (1:1,000; antibody sc-81178). The target proteins were visualized with a chemiluminescence reagent (PerkinElmer, MA, USA). The densities of the protein bands were individually normalized to the density of the actin band using ImageJ software. The reduced or increased levels of protein expression relative to the levels of expression for the control, which was set at 1.00, were determined.

### RNA extraction and real-time PCR.

Total RNA was isolated with the TRIzol reagent (Ambion, Life Technologies), and 2 μg of total RNA was used to synthesize cDNA using ImProm-II reverse transcriptase (Promega) according to the manufacturer's instructions. Real-time quantitative PCR (RT-qPCR) was performed using SYBR green mix (Life Technologies) on an Applied Biosystems QuantStudio 3 real-time PCR system. RT-qPCR was performed by using the following primer pairs: for GAPDH (glyceraldehyde-3-phosphate dehydrogenase), 5′-GATTCCACCCATGGCAAATTCCA-3′ (forward) and 5′-TGGTGATGGGATTTCCATTGATGA-3′ (reverse); for EV-A71, 5′-GCAGCCCAAAAGAACTTCAC-3′ and 5′-ATTTCAGCAGCTTGGAGTGC-3′; and for Hsp27, 5′-CACGAGGAGCGGCAGGAC-3′ (forward) and 5′-GGACAGGGAGGAGGAAACTTGG-3′ (reverse). Each sample was assayed in triplicate, and the gene for GAPDH was used as a reference gene. We analyzed the relative quantification of each gene using the 2^−ΔΔ^*^CT^* threshold cycle (*C_T_*) method.

### RNA immunoprecipitation.

Wild-type and Hsp27-KO RD cells were infected with EV-A71 at an MOI of 10 for 6 h and collected with lysis buffer (50 mM Tris-HCl, pH 7.4, with 150 mM NaCl, 1 mM EDTA, and 1% NP-40). Cell extracts were precleared with protein G-agarose for 1 h at 4°C to remove nonspecific complexes. Then, the precleared cell extracts were incubated with hnRNP A1 antibody and normal IgG as a negative control at 4°C overnight. The immunoprecipitation complex was pelleted by centrifugation at 1,000 × *g* for 5 min and washed eight times with lysis buffer. The pellet was resuspended in 400 μl of proteinase K buffer (100 mM Tris-HCl [pH 7.5], 12.5 mM EDTA, 150 mM NaCl, 1% SDS) and incubated with 100 μg of predigested proteinase K for 30 min at 37°C. The binding RNA was extracted with the TRIzol reagent and assessed by RT-qPCR. The PCR product was subjected to agarose gel electrophoresis.

### Immunofluorescence analysis.

We followed a previously reported immunofluorescence analysis protocol to study the hnRNP A1 distribution (30, 54). In brief, wild-type and Hsp27-KO RD cells were infected by EV-A71 at an MOI of 20 or 40 for 6 h. The cells were fixed with 4% paraformaldehyde for 20 min at room temperature, followed by permeabilization with 0.3% Triton X-100 in PBS. The cells were blocked with 5% BSA in PBS for 2 h and stained by incubation with anti-hnRNP A1 antibody (antibody sc-32301) and then with Alexa Fluor 488-conjugated anti-mouse immunoglobulin antibody. The nuclei were stained with DAPI (4′,6-diamidino-2-phenylindole) for 5 min. The images were captured on a Zeiss laser scanning microscope (LSM 880 NLO with Airyscan; Germany).

### Statistical analysis.

Results are expressed as the mean ± standard deviation (SD) in this study. All statistical analyses were carried out with SPSS (version 14.0) software (SPSS Inc.). A two-tailed Student's *t* test was applied for two-group comparisons. A *P* value of <0.05 was considered statistically significant.
